# Computational health economics for identification of unprofitable health care enrollees

**DOI:** 10.1093/biostatistics/kxx012

**Published:** 2017-03-22

**Authors:** Sherri Rose, Savannah L. Bergquist, Timothy J. Layton

**Affiliations:** * *Department of Health Care Policy, Harvard Medical School, 180 Longwood Ave, Boston, MA, 02115, USA* rose@hcp.med.harvard.edu

**Keywords:** Classification and prediction, Ensembles, Machine learning, Statistical methods in health economics, Variable selection

## Abstract

Health insurers may attempt to design their health plans to attract profitable enrollees while deterring unprofitable ones. Such insurers would not be delivering socially efficient levels of care by providing health plans that maximize societal benefit, but rather intentionally distorting plan benefits to avoid high-cost enrollees, potentially to the detriment of health and efficiency. In this work, we focus on a specific component of health plan design at risk for health insurer distortion in the Health Insurance Marketplaces: the prescription drug formulary. We introduce an ensembled machine learning function to determine whether drug utilization variables are predictive of a new measure of enrollee unprofitability we derive, and thus vulnerable to distortions by insurers. Our implementation also contains a unique application-specific variable selection tool. This study demonstrates that super learning is effective in extracting the relevant signal for this prediction problem, and that a small number of drug variables can be used to identify unprofitable enrollees. The results are both encouraging and concerning. While risk adjustment appears to have been reasonably successful at weakening the relationship between therapeutic-class-specific drug utilization and unprofitability, some classes remain predictive of insurer losses. The vulnerable enrollees whose prescription drug regimens include drugs in these classes may need special protection from regulators in health insurance market design.

## 1. Introduction

It is widely recognized by economists, health care providers, and policymakers that health insurance markets suffer from *adverse selection*. Often, a particular type of adverse selection based on consumer behavior is emphasized, where the tendency of sicker consumers to enroll in more comprehensive insurance plans drives up the price of more comprehensive plans and forces healthier consumers out of those plans. However, adverse selection can also present itself in the behavior of insurers, with insurers designing their health plans’ benefits to be attractive to healthy consumers and unattractive to sick ones. In the economics literature, this type of benefit distortion is termed service-level selection (Frank *and others*, 2000; Glazer and McGuire, 2000).

Due to the potential for adverse selection problems, many health insurance markets, including the state and federal Health Insurance Marketplaces created by The Patient Protection and Affordable Care Act (ACA), implement a policy known as “risk adjustment”, where insurers with sicker enrollees receive financial transfers from insurers with healthier enrollees. Risk adjustment causes the profitability of a particular enrollee to be less strongly correlated with the enrollee’s expected cost, because sicker enrollees with higher expected costs also generate higher revenues. Risk adjustment is far from perfect, however, potentially leaving unprofitable groups for insurers to identify and avoid (McGuire *and others*, 2014).

In this article, we take the hypothetical role of a profit-maximizing health insurer attempting to design its health plans (in conjunction with pharmacy benefit managers) to attract profitable enrollees and deter unprofitable ones. Such an insurer would not be acting in the interests of providing socially efficient levels of care by offering plans that maximize the overall benefit to society, but rather they would intentionally distort plan benefits in order to avoid high-cost enrollees to the possible detriment of both health and efficiency. In an ideal world, insurers would compete on the basis of quality and efficiency of care, not on their ability to identify and avoid unprofitable enrollees. But in competitive insurance markets, insurer incentives are typically consistent with inefficient selection-related, in addition to socially beneficial quality- or efficiency-related, competition (Glazer and McGuire, 2000). With the adoption of machine learning techniques across the health sector, there is unfortunately new potential to isolate novel relationships between enrollee characteristics and unprofitability in large health insurance enrollment and claims data, potentially exacerbating these types of selection-related behaviors. The work in this article is designed to provide transparency and discussion around this issue, and highlight vulnerable unprofitable groups that may need special protection from policymakers and regulators in health insurance market design.

We focus on a specific component of health plan design: the prescription drug formulary. In the new state and federal Health Insurance Marketplaces, the drug formulary is one of the most important dimensions on which insurers can distort their plan benefits in response to selection incentives. While other dimensions of health plan design are currently highly regulated (e.g., pre-existing conditions), insurers are effectively free to use the drug formulary to raise or lower the out of pocket cost of drugs used by people with a specific condition, thus allowing the insurer to make their formularies (and their health plans) more or less attractive to profitable or unprofitable groups. There is suggestive evidence of this type of behavior among insurers competing in the Health Insurance Marketplaces (Jacobs and Sommers, 2015; Geruso *and others*, 2017) and in Medicare Part D (Carey, 2017). These formulary distortions are likely to make it more difficult for unprofitable groups to find health plans that provide acceptable coverage for the drugs they take. It is also important to note that with anticipated changes to the Health Insurance Marketplaces through complete or partial repeal of the ACA, the weakening of protections for those with pre-existing conditions may be likely. One bill currently being discussed would permit insurers to charge high premiums for those with pre-existing conditions, and none of the four bills that have surfaced for congressional committee review provide a risk adjustment system.

We study the insurer’s incentive to distort coverage for drugs in a particular therapeutic class. This incentive is related to whether taking a drug in a given therapeutic class is predictive of a consumer’s profitability to the insurer. If consumers who use drugs in a given therapeutic class are unprofitable on average, then the insurer will want to weaken coverage for drugs in that class, either by placing those drugs on a formulary tier with high cost sharing or by removing most drugs in the class from the formulary altogether.

Our analytic approach centers around an ensembled machine learning method to determine which drug classes are most predictive of unprofitability, and thus most vulnerable to distortions by insurers in the Health Insurance Marketplaces. We implement an ensembling framework that selects the optimal weighted combination among all considered algorithms in a super learner (van der Laan *and others*, 2007) to build the prediction function. The best ensemble is chosen with respect to minimizing the squared error loss function, evaluating each candidate weighted combination of algorithms based on cross-validated mean squared error. Thus, this framework allows application of multiple algorithms (eliminating the need to guess beforehand which single algorithm might perform best in the given data) with the opportunity to outperform any single algorithm by additionally considering all weighted averages of algorithms. Our implementation also includes an application-specific variable selection approach designed for this study that combines both data-adaptive techniques and investigator knowledge, all defined a priori. The super learner has been used in previous works within health care to predict post-traumatic stress disorder based on traumatic experiences (Kessler *and others*, 2014), predict mortality in intensive care units (Pirracchio *and others*, 2015), and to develop health plan payment risk adjustment formulas for total annual health care expenditures (Rose, 2016), among other applications.

## 2. Cohort

Potential health insurance enrollees seeking plans through the Health Insurance Marketplaces, as defined by the ACA, include those who are: (i) uninsured or (ii) insured with a non-group or small group insurance policy. Currently, however, data on individuals enrolled in large group health insurance plans are used by the federal government to inform decision-making for Health Insurance Marketplace enrollees. The Truven MarketScan database is a longitudinal enrollment and claims database from large employers and insurers containing up to 51 million enrollees per year (Adamson *and others*, 2008). Truven MarketScan data from 2010 were used to calibrate the federal risk adjustment system for the Health Insurance Marketplaces beginning in 2014, and are likely to be used for any recalibration in 2017 and beyond. This is problematic as the Health Insurance Marketplace and commercial Truven MarketScan populations are fundamentally different with respect to age, health status, and other characteristics. Thus, while we also use Truven MarketScan data, we follow earlier literature (Rose *and others*, 2015; Layton *and others*, 2015) to select a sample that is more representative of Health Insurance Marketplaces enrollees.

### 2.1. Cohort selection

Our inclusion and exclusion criteria mirrored those used by the federal government in 2014 for the Health Insurance Marketplace risk adjustment formulas (Kautter *and others*, 2014). Therefore, enrollees are in a preferred provider organization or other fee-for-service health plan in both the first and last month of each year considered, do not make capitated payments, are age 21–64, and have mental health and drug coverage. Individuals with negative payments for services were removed. A total of 7 072 964 individuals met these criteria for 2012–2013. Using propensity-score techniques described in previous literature (Layton *and others*, 2015), we identified the 2 006 216 observations that met our criteria to be representative of the Health Insurance Marketplace population with regard to distributions of age, gender, region, residence in a metropolitan statistical area, inpatient admissions, number of inpatient admissions, and quantile of outpatient and prescription drug spending.

### 2.2. Data extraction

Our analytic dataset focuses entirely on prescription drug related variables. Included are indicators for identifying drugs as single-source brand, multi- or single-source generic, or over the counter products; indicators classifying drugs as primarily for long-term treatment of chronic conditions, primarily for short-term treatment of acute conditions, or used for both; therapeutic class indicators based on the therapeutic/pharmacological category; and therapeutic group indicators, which are aggregations of the therapeutic class indicators (Adamson *and others*, 2008). The therapeutic classes are based on 2008 RED BOOK codes (Thomson Healthcare, 2008). Thirteen of the therapeutic groups are composed of a single therapeutic class; after dropping these and the 48 therapeutic indicator variables for which there are no positive claims in our sample, we have 239 binary drug-related variables to predict unprofitability. We specifically do not use variables such as age given they are already controlled for in an individual insurance market like the Health Insurance Marketplaces through risk adjustment.

### 2.3. Feature choices

We define individual-level insurer profits $F_i$ as being equal to revenues minus costs:

$$F_{i}=R_{i}-C_{i}.$$

We define costs as the sum of all health care spending (inpatient, outpatient, and prescription drug) for person $i$ in a given year and observe this value in our data. Revenues are not observed directly in the data but can be derived. We calculate revenues according to Marketplace plan payment formulas specified by the Secretary of Health and Human Services (HHS). Marketplace plan revenues consist of two components: premiums, $M_i$ and risk adjustment transfers, $A_i$. Regulation allows limited variation in premiums across individuals based on age, geography, and smoking status. We follow previous literature by abstracting from age-, geography-, and smoking status-based premium variation and assuming that competition forces all plans to charge a premium equal to the average cost in the market (Layton *and others*, 2015):

$$M_{i}=\bar{C}=\frac{1}{n}\sum_{i=1}^{n}C_{i},$$

for all $i$. For risk adjustment transfers, we start by specifying a risk score, $S_i$, for each individual using the risk adjustment formula used in the Marketplaces (Kautter *and others*, 2014). This formula assigns risk scores according to diagnoses in claims data. We use an individual’s diagnoses from 2013 to assign their risk score. We then specify risk adjustment transfers according to a simplified version of the Marketplace risk adjustment transfer formula:

$$A_{i}=(\frac{S_{i}}{\bar{S}}-1)\bar{C},$$

where $\bar{S}=\frac{1}{n}\sum^n_{i=1}S_i$. Given these two components, we can then generate calculated revenues, and thus profits, at the individual level:

$$\begin{array}{cc} F_{i} & \;=M_{i}+A_{i}-C_{i}\\ & \;=\bar{C}+(\frac{S_{i}}{\bar{S}}-1)\bar{C}-C_{i}\\ & \;=\bar{C}\times \frac{S_{i}}{\bar{S}}-C_{i}. \end{array}$$

Given that we are interested in predicting *unprofitability*, our understanding of individual-level insurer profits allows us to easily define individual-level insurer unprofitability $U_i$ as costs minus revenue:

$$U_{i}=-F_{i}=C_{i}-R_{i}.$$

We can also now compute unprofitability at the individual level:

$$\begin{array}{cc} U_{i}=-F_{i} & \;=C_{i}-M_{i}-A_{i}\\ & \;=C_{i}-\bar{C}-(\frac{S_{i}}{\bar{S}}-1)\bar{C}\\ & \;=C_{i}-\bar{C}\times \frac{S_{i}}{\bar{S}}. \end{array}$$

## 3. Methods

The super learner is a general ensembling framework that can be applied to build a prediction function that is the optimal weighted combination of a library of algorithms (van der Laan *and others*, 2007). The general principle is that, by positing a family of weighted combinations of algorithms, one of these weighted algorithms may outperform any given single algorithm in the library. The theory of the super learner is described in previous literature (van der Laan and Dudoit, 2003; van der Laan *and others*, 2007), which presents both asymptotic and finite sample properties that guarantee the super learner approximates the unknown oracle selector (i.e., the best weighted combination among included algorithms). Earlier work includes stacking algorithms (Wolpert, 1992; Breiman, 1996; LeBlanc and Tibshirani, 1996). The super learner generalized stacking and provided new optimality properties. Individual algorithms within the super learner may have different tuning parameters (e.g., random forests with 500 trees and random forests with 1000 trees would be unique algorithms) and consider differing sets of variables.

### 3.1. Algorithm

Consider our outcome $U$, unprofitability, and a vector of all drug-related variables $X$. The observational unit is described by $O=(U,X)$, drawn from unknown true probability distribution $P_0$, where we measure $U_i$ and $X_i$ for each enrollee. We additionally specify a nonparametric model $\mathcal{M}$ that is a collection of possible probability distributions $P$. We assume only that our data are $n$ independent and identically distributed draws of random variable $O$. The parameter of interest is

$$\Psi (P_{0})=E_{0}(U|X)={\text{arg min}}_{\Psi (P)}E_{0}L(O,\Psi (P)),$$

where the loss function $L$, which takes as input the observed data $O$ and candidate functions $\Psi(P)$, is the squared error loss: $L(O,\Psi(P))=(U-\Psi(P))^2$. Minimizing the expected loss $E_0L(O,\Psi(P))$ yields the true conditional mean $E_0(U\mid X)$. While $\Psi(P_0)$ is unknown, we seek the best estimator of this conditional mean. We could have considered other loss functions or transformed our unprofitability outcome measure with a log modification. However, the health spending literature indicates that raw costs perform well for prediction compared to other choices (Dunn *and others*, 2003; Jones *and others*, 2007; Ellis *and others*, 2017).

The super learner for $\Psi(P_0)=E_0(U\mid X)$ is constructed as follows:

1. Fit each of $K$ candidate algorithms within $V$-fold cross-validation. This involves dividing the dataset $O$ into a training set containing $\frac{V-1}{V}^{\text{ths}}$ of the data and a validation set containing the remaining $\frac{1}{V}^{\text{th}}$of the data in each of $V$ folds. The training set $T(v)$, $v=1,\ldots,V$, is used to generate the algorithm fit in each fold while the $V(v)$ validation set is then fed through the fitted algorithm to obtain cross-validated predicted values $Z_k(v)$.
2. Posit a family of weighted combinations of the $K$ algorithms that is a convex combination indexed by $\alpha$, and select the $\hat\alpha$ that minimizes the expected loss. This reduces to a simple minimization problem where we regress $U$ on $Z$:
  $$E(U|Z)=\alpha_{1}Z_{1}+\ldots +\alpha_{K}Z_{K}.$$
3. Run all $K$ algorithms on the full data $O$ and combine the candidate fits $\hat{\Psi}(P)$ with the $\hat\alpha$ vector to build the super learner function:
  $$\hat{\Psi }(P{)}_{SL}={\hat{\alpha }}_{1}\hat{\Psi }(P{)}_{1}+\ldots +{\hat{\alpha }}_{K}\hat{\Psi }(P{)}_{K},$$
  and obtain final predicted values.

### 3.2. Implementation

Beyond including a diverse collection of algorithms in our library, it is also often of interest to consider differing sets of predictors. If an exhaustive set of variables does not have better predictive performance than a smaller subset, chosen either data-adaptively or via subject matter knowledge or both, it may be beneficial to implement a final algorithm using the smallest set necessary. Thus, our implementation incorporates three different variable sets. The full variable set contains all 239 binary drug variables; a more parsimonious set includes 31 therapeutic group indicators that aggregate the drug variables into hierarchies, plus eight generic indicators and five long-term and short-term use indicators; and the final set is a small selection of variables chosen data-adaptively by an application-specific lasso penalized regression within the cross-validation folds. We limited this new implementation of lasso regression to ten non-zero variables. When the number of non-zero coefficients exceeded ten, a larger value of the regularization parameter $\lambda$ was used to select a smaller set of variables excluding the ties. Additionally, and most importantly, we augmented the subset of variables chosen by the lasso to include indicators for the therapeutic classes of HIV and multiple sclerosis drugs (if they were not already selected) given the high cost of these therapies. We select a set of approximately 10 variables based on prior work demonstrating that ten variables chosen data-adaptively can be nearly as predictive as a larger set of variables for total health spending (Rose, 2016).

The five distinct algorithms that consider each of these three variable sets are a neural network with two units in the hidden layer, lasso penalized regression with the $\lambda$ value chosen via internal cross-validation, ridge regression with the $\lambda$ value chosen via internal cross-validation, a regression tree with ANOVA splitting, and a main-terms linear regression. This led to a total of 15 individual algorithms ensembled by the super learner ($K=15$) using 10-fold cross-validation. Our implementation relied on the SuperLearner package in the R programming language, which also called the nnet, glmnet, and rpart packages (Polley and van der Laan, 2013; Friedman *and others*, 2016; Ripley and Venables, 2016; Therneau *and others*, 2015). Our unique application-specific implementation of the lasso regression to screen variables described above is freely available as R code (see Section 6).

### 3.3. Evaluation approach

We evaluate the performance of the super learner with respect to the a priori selected loss function $L$, the squared error loss. Thus, using the cross-validated predicted values, we construct cross-validated mean squared errors:

$${\text{CV MSE}}_{k}=\frac{\sum_{i=1}^{n}(U_{i}-Z_{k,i}{)}^{2}}{n},$$

and cross-validated $R^2$ values:

$$\text{CV}R_{k}^{2}=1-\frac{\sum_{i=1}^{n}(U_{i}-Z_{k,i}{)}^{2}}{\sum_{i=1}^{n}(U_{i}-\bar{U}{)}^{2}},$$

for each individual algorithm $k$ considered, as well as the super learner. Note that in order to obtain a cross-validated mean squared error and cross-validated $R^2$ for the super learner, the *entire* procedure described in Section 3.1 is itself cross-validated with 10-fold cross-validation.

## 4. Predicting unprofitability results

**Fig. 1.**
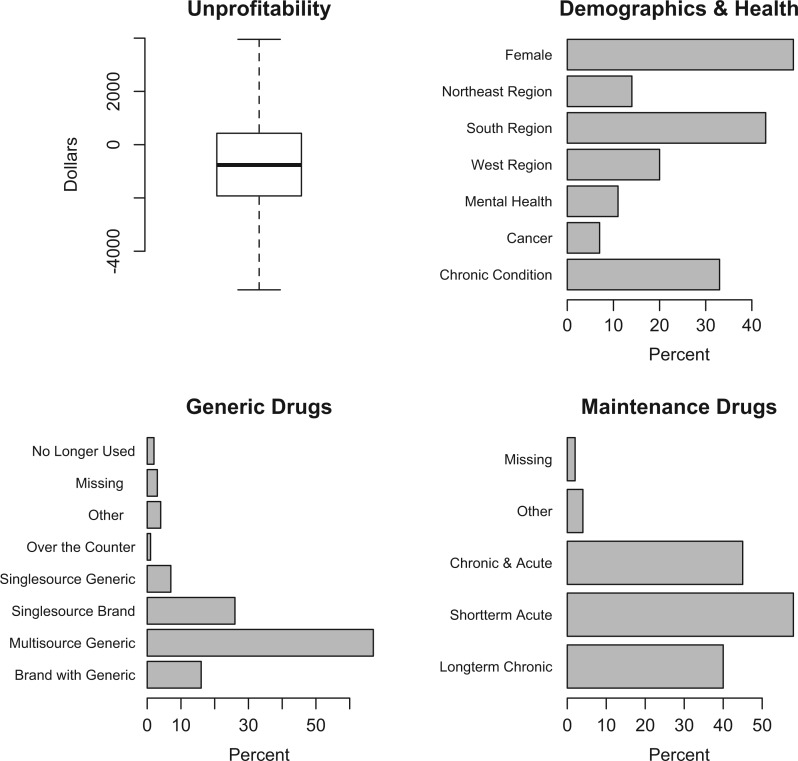
Summary values for unprofitability, demographics and health, generic drugs, and maintenance drugs. Outliers were dropped from the unprofitability plot.

Summary information for key variables in the Truven MarketScan data are described in Figure 1. The median value of unprofitability was $-\$762$ (indicating the median enrollee was not, in fact, unprofitable), with a mean of $\$0$ (standard deviation: $\$15 617$). Mean age was 42 years, 49% of our sample was female, and 33% of enrollees have one or more chronic conditions. The final super learner algorithm was defined by:

$$\hat{\Psi }(P{)}_{SL}=0.15\hat{\Psi }(P{)}_{\text{nnet.f}}+0.04\hat{\Psi }(P{)}_{\text{nnet.g}}+0.69\hat{\Psi }(P{)}_{\text{glm.f}}+0.03\hat{\Psi }(P{)}_{\text{glm.g}}+0.09\hat{\Psi }(P{)}_{\text{glm.l}},$$

where nnet is the neural network algorithm and glm is the main terms linear regression, and the appendices .f, .g, and .l indicate the full set of covariates, therapeutic groups only, and the application-specific lasso, respectively.

**Fig. 2.**
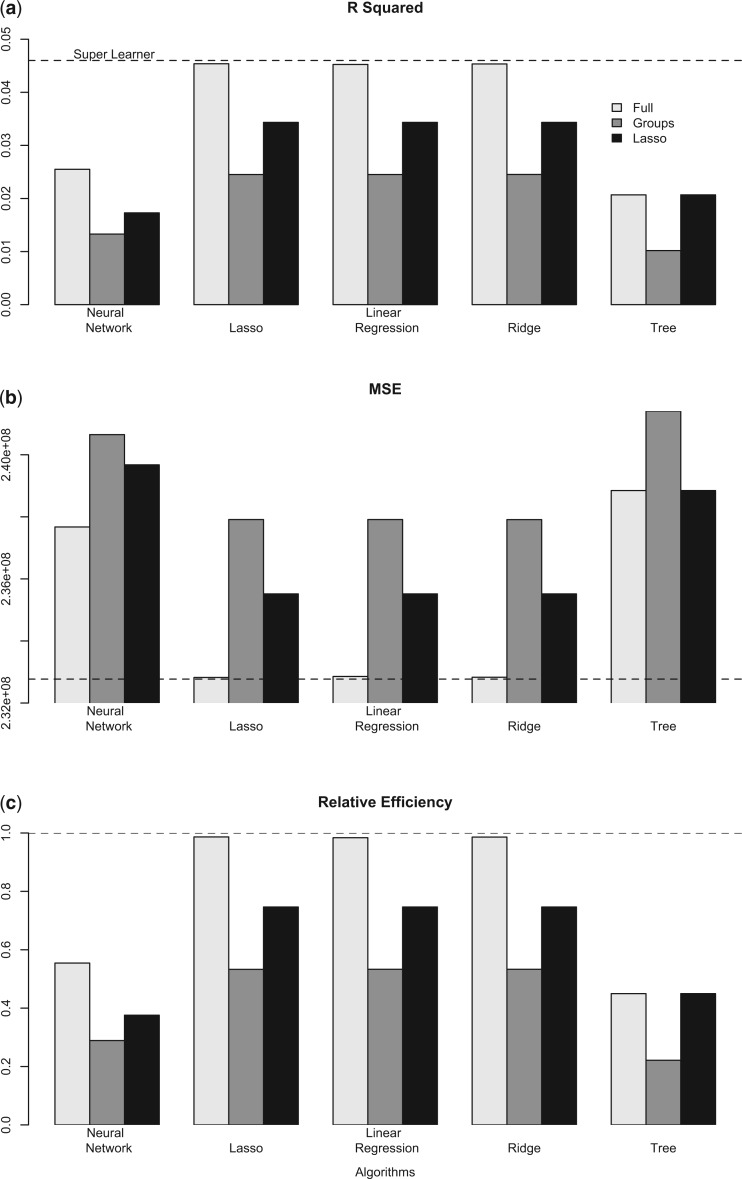
Results from unprofitability algorithms with varying variable subgroups. (a) Cross-validated $R^2$, (b) Cross-validated mean squared error, (c) Cross-validated relative efficiency, where relative efficiency = CV$R^2_{\texttt{k}}$/CV$R^2_{\texttt{SL}}$. Dashed line is performance of super learner.

We found that drug classes had signal for predicting unprofitability (Figure 2), with a cross-validated $R^2$ of 4.6% for the super learner. The Marketplace risk adjustment system accounts for a large portion of the variation in spending. Our result may suggest that the system is working reasonably well at matching revenues to expected costs, although still leaving room for drug utilization to be exploited. Drug spending is responsible for less than 20% of total spending and around 30% of individuals in our sample have no drug spending, so, while a cross-validated $R^2$ of 4.6% may seem low compared to other applications, this is a stronger signal than we should ideally see. We explore this issue further in Section 5. Additionally, we performed a falsification test with an unrelated simulated outcome measure, and obtained a cross-validated $R^2$ of 0.0%.

The super learner had the best overall performance, with the largest cross-validated $R^2$ (Figure 2a), and the smallest cross-validated mean squared error among all algorithms considered (Figure 2b). However, its improvement over the best single algorithm was minimal, where the lasso algorithm using the full covariate set had a relative efficiency of 98.7% (Figure 2c). The ridge regression and the linear regression, both with the full covariate set, also had relative efficiencies close to 1. The worst performing algorithm was the regression tree using the therapeutic groups only variable subset, with a cross-validated $R^2=1.0\%$ and a relative efficiency of 22.2% compared to the super learner. Overall, the regression tree and neural network had poorer performance within each variable subset compared to the two penalized regressions and the linear regressions, with relative efficiencies ranging from 22.2 to 55.4% vs. 53.3 to 98.7%.

The more parsimonious set of therapeutic groups was less predictive of unprofitability than the full set of variables. The relative efficiency of the algorithms with the therapeutic groups subset compared to the super learner ranged from 22.2 to 53.3%. The subset of variables chosen by the application-specific lasso variable selector included two generic indicators (multi-source generic and over the counter) and eight therapeutic class indicators (macrolide antibiotics; cephalosporin antibiotics; quinolones [synthetic antibiotics]; antivirals, including HIV drugs; biological response modifiers, including multiple sclerosis drugs; anti-inflammatory agents for ear, eye, nose, and throat; fluoride preparations; and calcium supplements for replacement preparation). Notably, the small set of variables chosen by the lasso was more predictive than the subset containing the therapeutic groups for all algorithms, and for the regression tree, the lasso subset had an identical cross-validated MSE compared to the full set. The relative efficiency of the algorithms with the lasso subset compared to the super learner ranged from 37.5 to 74.7%.

We also calculated the difference between the actual values for unprofitability and the predicted values generated by the super learner algorithm. While these differences were clustered around zero (results not shown), there were three obvious extreme outliers. The observed drug utilization patterns for these outliers reflect those seen in individuals who are unprofitable, but on the order of $\$10$–35K versus their actual $\$2.5$–3.7M. It is well known that there are typically extreme outliers when it comes to health care spending, where even including additional variables beyond drug utilization would not have likely dramatically improved these predictions (Ellis *and others*, 2017).

## 5. Discussion

The ability of health insurers to distort offerings in their drug formularies is an area of rising concern in the Health Insurance Marketplaces. There are currently strict regulations protecting other dimensions of health plan design, but not the drug formularies. The fact that health insurers cannot discriminate based on pre-existing conditions in the Health Insurance Marketplaces (as they exist today) is a major component of the ACA, and has made premiums affordable to those without group insurance. However, drug formularies remain potentially gameable. A profit-maximizing insurer may have incentives to use them to avoid unprofitable enrollees as risk adjustment imperfectly controls for their health. Potential “repeal and replace” or “repeal and repair” of the ACA would likely lead to fewer protections for individuals with pre-existing conditions, including allowing insurers to charge unaffordable premiums based on these health conditions. It is estimated that *at least* 5.4 million of the 20 million individuals currently enrolled in insurance through the Health Insurance Marketplaces established by the ACA have pre-existing conditions (Claxton *and others*, 2016). Further discrimination through the drug formulary, as shown in our article, could remove additional enrollees from needed insurance and access to care.

We demonstrated that ensembled machine learning methods can be implemented to extract the remaining relevant signal from the drug formulary data in order to predict unprofitability. Our results are both encouraging and concerning. On one hand, the sophisticated learning algorithms used in this article produced functions that were not extremely highly predictive of unprofitability. This suggests that the risk adjustment system may be performing reasonably well at matching revenues to expected costs and limiting insurer incentives to distort drug formularies to attract healthy and deter sick enrollees. On the other hand, the algorithms were able to relate drug utilization to unprofitability for some therapeutic classes, implying that distortionary insurer incentives remain for these classes, and Marketplace enrollees who use drugs in these classes may find it difficult to find health insurance plans that provide adequate coverage for their prescribed drug regimens. Strikingly, the two generic indicators and eight therapeutic class indicators identified by our application-specific lasso maintained about 75% of the predictive performance of the full set of 239 drug-related variables. We also highlight that $R^2$ values for health spending applications are generally low, with values for total health spending prospective risk adjustment formulas ranging from 20 to 25%, Medicare Advantage formulas around 11 to 12%, and mental health care spending below 7% below 7%(Hermann *and others*, 2007; Pope *and others*, 2011; Ellis *and others*, 2017). These estimates also typically do not reflect cross-validated $R^2$, and are thus overestimates of the cross-validation-based metrics we use here.

As noted above, the subset of variables chosen by the lasso variable screener and provided to each of the individual algorithms included two generic indicators (multi-source generic and over the counter) and eight therapeutic class indicators for a wide range of drug types. Many of the therapeutic classes considered in the full variable set were similarly predictive of unprofitability, making it difficult for a regulator to protect consumers through targeting those individual drugs for protections. For example, Medicare’s Part D “protected classes” policy is intended to protect patient access to specific drugs (including HIV drugs) and to ensure that patients are not discouraged from enrolling in certain Part D plans (CMS, 2016). A protected classes approach may ensure that patients enrolling in a plan have access to the specified drugs, but it would not necessarily guard against more subtle “adverse tiering” behavior consisting of plans placing drugs used by unprofitable enrollees on formulary tiers with high levels of cost-sharing. Thus an insurer may wish to perform variable selection to determine which set of drugs are indicative of unprofitability, and to reduce coverage for these drug types.

While we played the role of a *hypothetical* profit-maximizing insurer, there are suggestive indications that this may be occurring in practice, as well as a new lawsuit asserting it *is* happening. Carey (2017) shows that in the Medicare Part D prescription drug insurance market, insurers respond to the selection incentives we highlight here. In her article, she shows that Part D plans offer less generous coverage for drugs used by unprofitable groups. Jacobs and Sommers (2015) show that some plans competing in the state and federal Health Insurance Marketplaces also use drug formularies to avoid individuals with HIV. They find that individuals with HIV who enrolled in these “Adverse-Tiering Plans” would have an average annual cost per drug of more than triple the cost they would have faced in a “non-Adverse-Tiering Plan.” Notably, a recent lawsuit filed by Harvard Law School in September 2016 asserts that major insurers in the Health Insurance Marketplaces *are* designing their coverage so that individuals with HIV/AIDS will be less likely to enroll (Gorenstein, 2016).

There are also employment trends for deep analytic talent within the insurance space that imply health companies are looking to extract as much information as possible from their data. The broad view of these trends within the United States was captured in a McKinsey study (Manyika *and others*, 2011). They reported that, of 152 900 workers with deep analytic skills, 22 300 (14.5%) were employed by insurance carriers, agencies, brokerages, and other insurance-related companies. Remarkably, 9600 of those in this group were *not* actuaries, with 3200 statisticians. While not all of these employees are in the health sector, we also have anecdotal evidence that health insurers are building strong data-centric teams. For example, one major health insurer noted last year that they hire talent from other sectors that adopted data science tools earlier than health care, such as retail and financial technology, to work in their analytics department (Chuang, 2016).

The super learner framework provides researchers with the opportunity to run many algorithms, an honest assessment of performance, and considers all weighted averages of the candidate algorithms. This is at effectively no computational cost, as after cross-validation is performed, the estimation of the weight vector is a simple linear regression of the outcome on the cross-validated predicted values. Thus, even when the super learner has only moderately improved performance compared to any single algorithm, it may still be valuable to allow for any improvement provided by a weighted average. However, in clinical or policy settings, simplicity may be preferred. In these cases, researchers should include the standard practice approach, which may be a very small number of variables in a main-terms regression or “checklist” classification algorithm, in the super learner. An a priori threshold for improvement can then be set, such that only when any single algorithm or the super learner outperforms the standard approach by a preset amount would that prediction function be defined as best. Earlier studies have shown that super learner can have substantially better performance than any single algorithm in other applied analyses (van der Laan and Rose, 2011). The super learner framework also allows investigators to consider varying sets of variables within algorithms, which was a strategic advantage here.

This is the first study to examine the predictability of unprofitability with drug variables using ensembles or any other technique. The goal of this work was to explore the ability of insurers to use this data to identify potentially vulnerable unprofitable groups. In future work, we are comparing similar measures of insurer incentives to distort drug formularies to actual coverage by therapeutic class among plans competing in the state and federal Health Insurance Marketplaces. This research will allow us to know not only what selection-related incentives insurers face but whether they’re acting on those incentives, potentially providing additional motivation for policymakers and regulators to act to provide insurers with incentives that are more consistent with social objectives.

## 6. Software

Simulated data and software in the form of publicly-available R code is online at: sl-bergquist.github.io/unprofits.

## Supplementary Material

Supplementary Data
