# Supplementary material for: Computational health economics for identification of unprofitable health care enrollees
Source: Biostatistics. 2017 Mar 22;18(4):682–94. doi: 10.1093/biostatistics/kxx012 (PMC5862318; doi:10.1093/biostatistics/kxx012)
Supplement: Supplementary Data [file kxx012_supp.pdf]

# Computational Health Economics for Identification of Unprofitable Health Care Enrollees

## Supplementary Materials

Sherri Rose, Savannah L. Bergquist, Timothy J. Layton
Department of Health Care Policy, Harvard Medical School

February 10, 2017

# Simulation Study and Code

The MarketScan data used in our analyses contains protected patient health claims information and is also proprietary, thus we are not able to share it online to reproduce our study. We therefore designed a simulated data set based on the MarketScan data, creating a population of 2,006,126 observations with binary covariates $X$ and an unprofitability outcome variable, $U$. Additionally, we sampled from this population to create a smaller data set of 250,000 observations. The 250,000 observation data set may be more suitable for users who wish to run the analysis code on a laptop. These data sets, and accompanying code, are available online sl-bergquist.github.io/unprofits.

## Variable Creation

The 247 covariates $X$ are representative in distribution to the covariates in our real data. The vector length of $X$ differs slightly in number from the 239 drug-related covariates $X$ in the manuscript due to random variability in the simulation related to whether a therapeutic indicator variable had positive claims. Therapeutic groups were aggregated based on the groupings of therapeutic class indicator variables contained in the MarketScan data documentation, as found in the 2008 RED BOOK (Thomson Healthcare, 2008). Additionally, we mimicked the correlation among the variables identifying the generic status of drugs and among the variables classifying drugs as for long- or short-term treatment of conditions, although dependence between these and other variables was simplified to maintain anonymity of the underlying data source. Our outcome variable, $U$, was drawn from a truncated normal distribution with a lower bound $a$ and an upper bound $b$:

$$U \sim Norm(b_U, 10411.37, a = -485100, b = 3743000),$$
$$b_U = \alpha + \beta_k\gamma_k + \beta_m\lambda_m + \beta_j X_j + \epsilon,$$

where $\gamma$ is a vector of $k$ variables identifying drugs as single-source brand, multi- or single-source generic, or over the counter products, $\lambda$ is a vector of $m$ variables identifying drugs as for the

long-term treatment of chronic conditions, the short-term treatment of acute conditions, or for both, and $X$ is a vector of $j$ therapeutic class indicators.

## Results

Using the 2,006,126 observation simulated data set, we obtained the results found in Figure 1. The results in our simulated data demonstrated similarities to our analysis of the MarketScan data, including lasso, linear regression, and ridge as the top three single-algorithm approaches, and the full covariate set providing the best performance for each single algorithm. The super learner function was given by:

$$\hat{\Psi}(P)_{SL} = 0.01\hat{\Psi}(P)_{\texttt{nnet.l}} + 0.28\hat{\Psi}(P)_{\texttt{lasso.f}} + 0.71\hat{\Psi}(P)_{\texttt{glm.f}},$$

where `nnet` is the neural network algorithm, `lasso` is the lasso, and `glm` is the main terms linear regression, and the appendices `.f` and `.l` indicate the full set of covariates and the application-specific lasso, respectively. This super learner had a cross-validated $R^2$ of 7.1%, and the single best algorithm, the main terms linear regression with the full set of variables, had a cross-validated $R^2$ of 7.1%. Here, the super learner performs as well as the best algorithm, whereas in our data analysis is does improve on the single best algorithm (there, it was the lasso). The simulated data analysis was performed with `R` version 3.3.1 and the `SuperLearner` package (Polley and van der Laan, 2013) on an Oracle Sun Server X4-4 with 60 cores and 1.5TB of RAM with Linux software.

# References

E. Polley and M.J. van der Laan. *SuperLearner: Super Learner Prediction*, 2013. URL `http://CRAN.R-project.org/package=SuperLearner`. R package version 2.0-15.

Thomson Healthcare. *RED BOOK: Pharmacy's Fundamental Reference.* Thomson Healthcare Red Book Drug Topics, 2008.

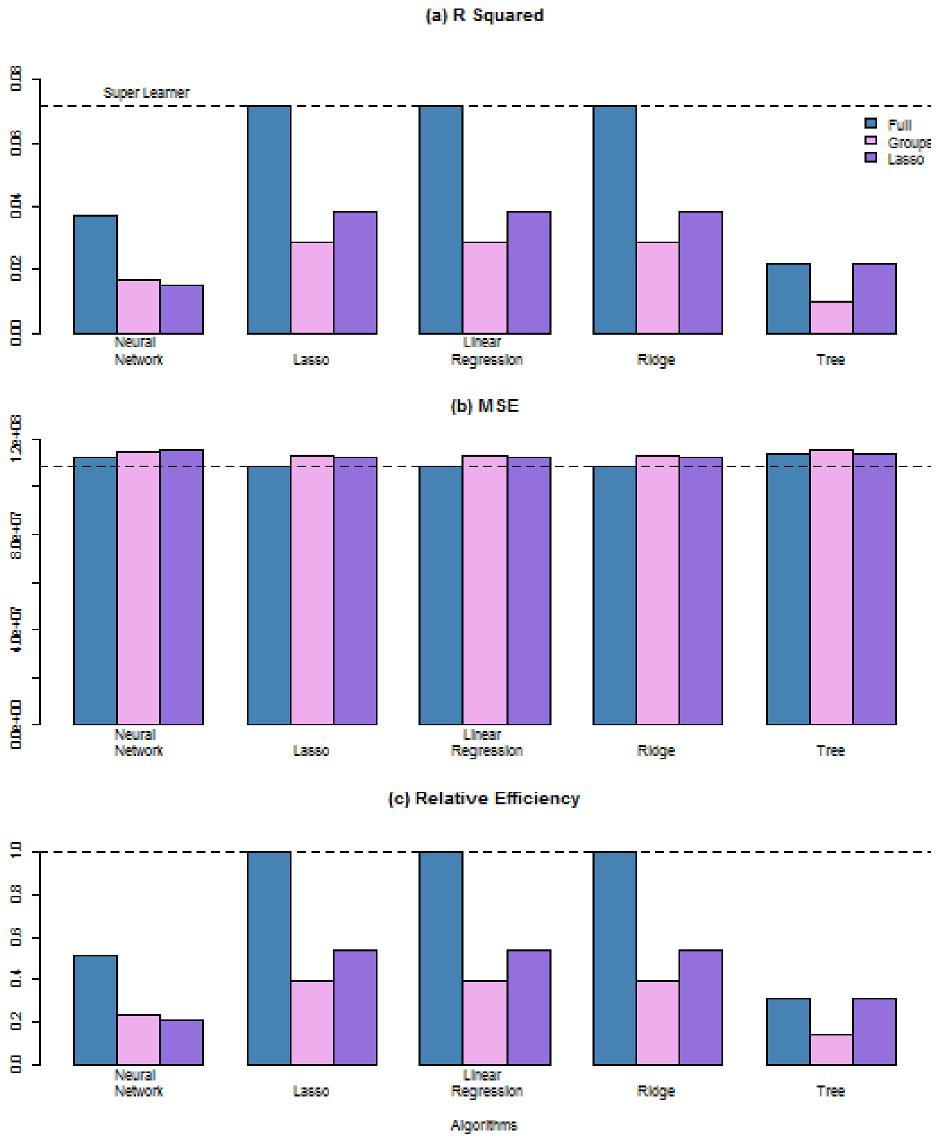


**Figure 1:** Results from unproftability algorithms with varying variable subgroups on simulated data set of 2,006,126 observations. (a) Cross-validated $R^2$,(b) Cross-validated mean squared error, (c) Cross-validated relative efficiency (CV$R_k^2$ / CV $R_{SL}^2$). Dashed line represents the performance of the super learner.
